# Supplementary material for: First Report of Distinct Bamboo mosaic virus (BaMV) Isolates Infecting Bambusa funghomii in Vietnam and the Identification of a Highly Variable Region in the BaMV Genome
Source: Viruses. 2022 Mar 28;14(4):698. doi: 10.3390/v14040698 (PMC9032891; doi:10.3390/v14040698)
Supplement: Supplementary file 1 [file viruses-14-00698-s001.zip › Figure S1.pptx]

## Slide 1
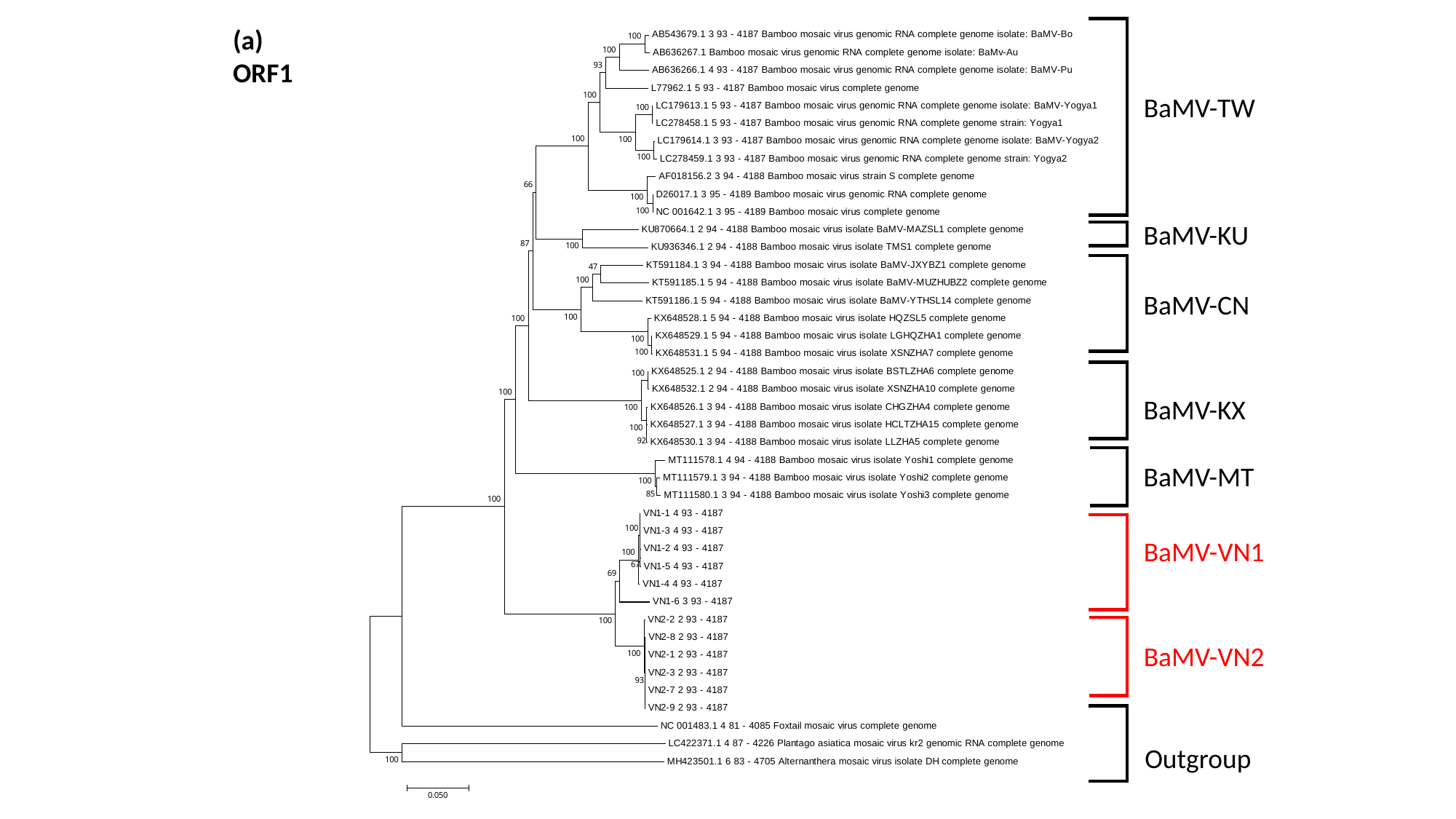

(a)
ORF1
BaMV-TW
BaMV-KU
BaMV-CN
BaMV-KX
BaMV-MT
BaMV-VN1
BaMV-VN2
Outgroup

## Slide 2
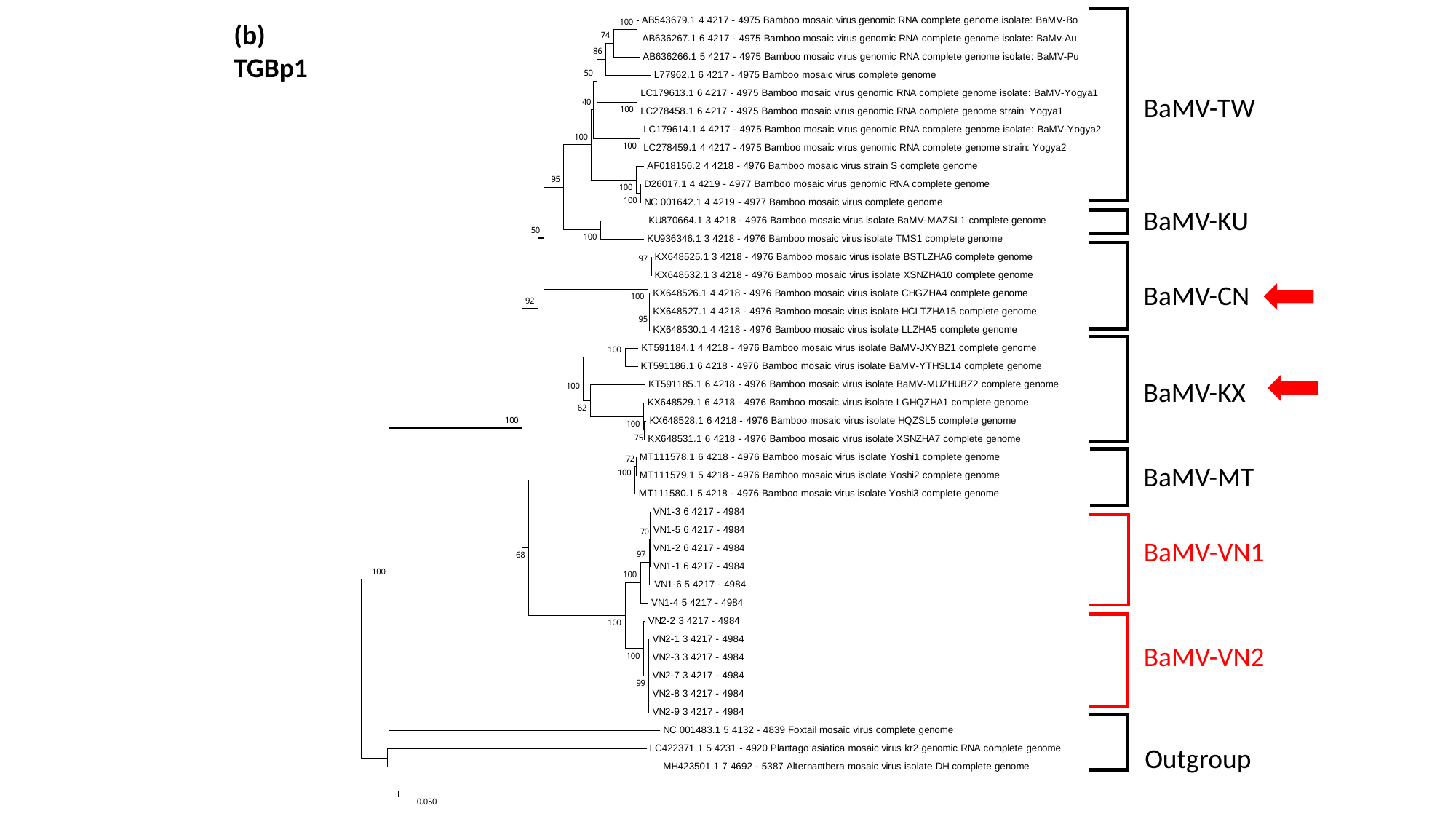

(b)
TGBp1
BaMV-TW
BaMV-KU
BaMV-CN
BaMV-KX
BaMV-MT
BaMV-VN1
BaMV-VN2
Outgroup

## Slide 3
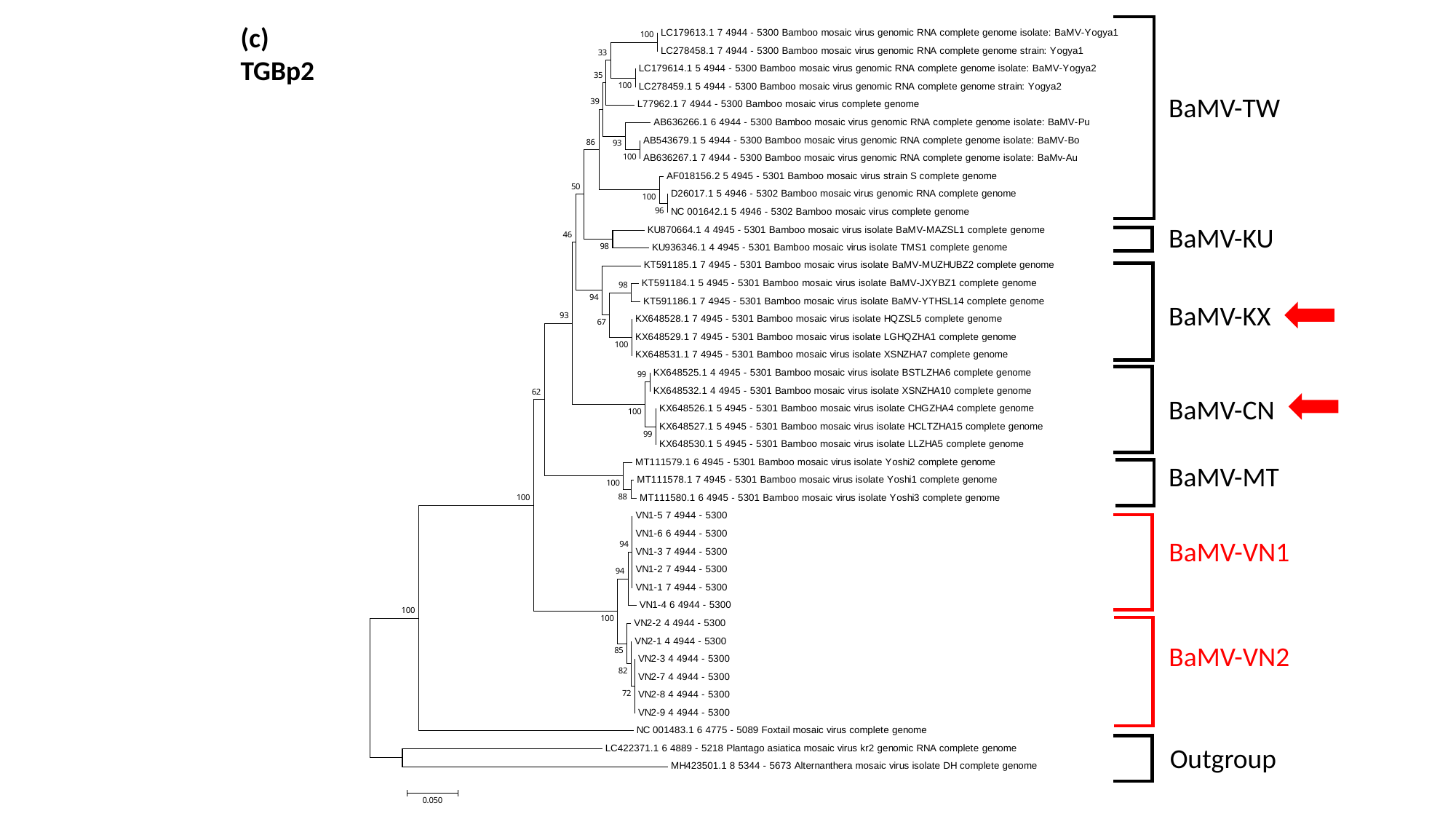

(c)
TGBp2
BaMV-TW
BaMV-KU
BaMV-KX
BaMV-CN
BaMV-MT
BaMV-VN1
BaMV-VN2
Outgroup

## Slide 4
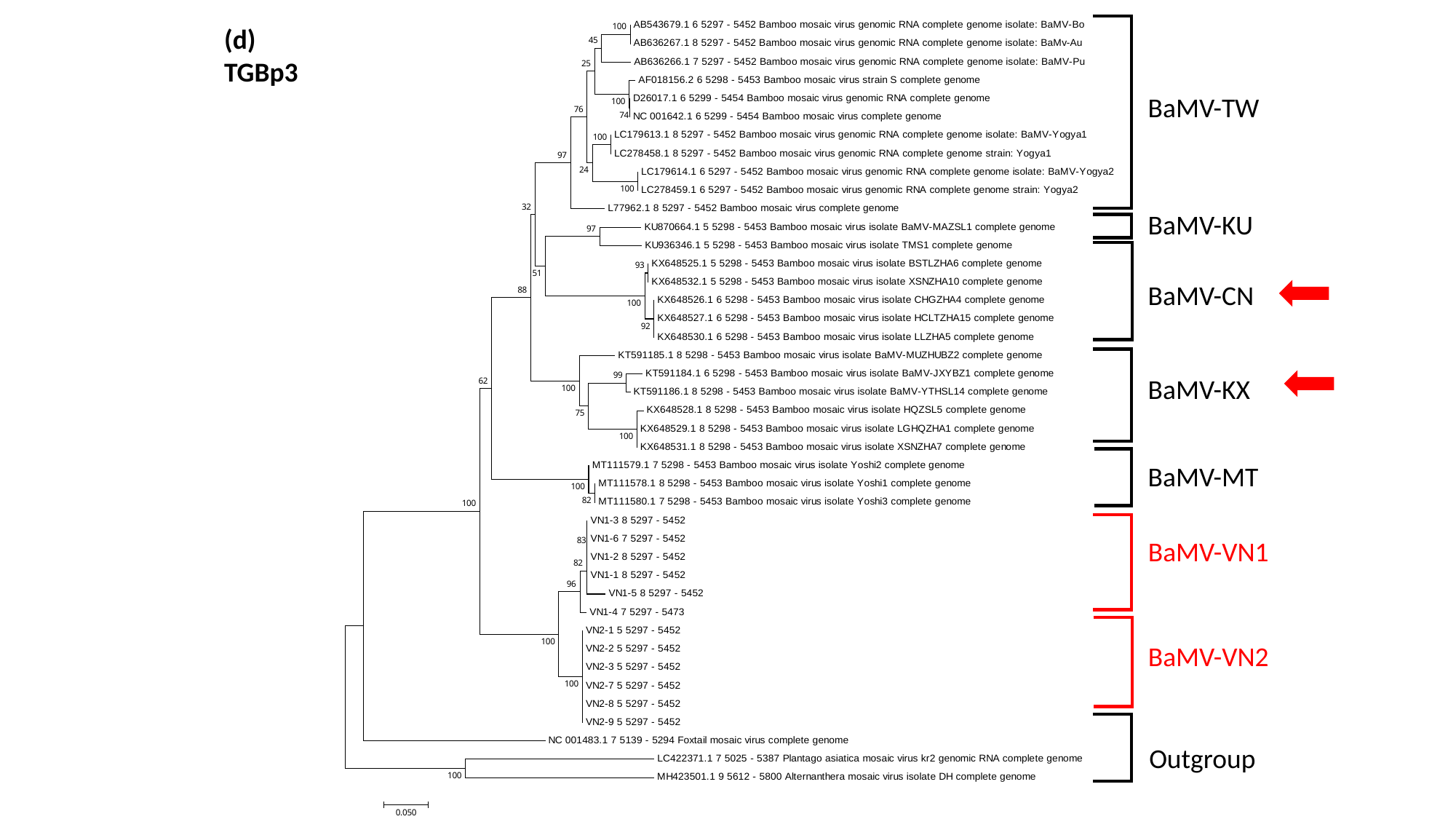

(d)
TGBp3
BaMV-TW
BaMV-KU
BaMV-CN
BaMV-KX
BaMV-MT
BaMV-VN1
BaMV-VN2
Outgroup

## Slide 5
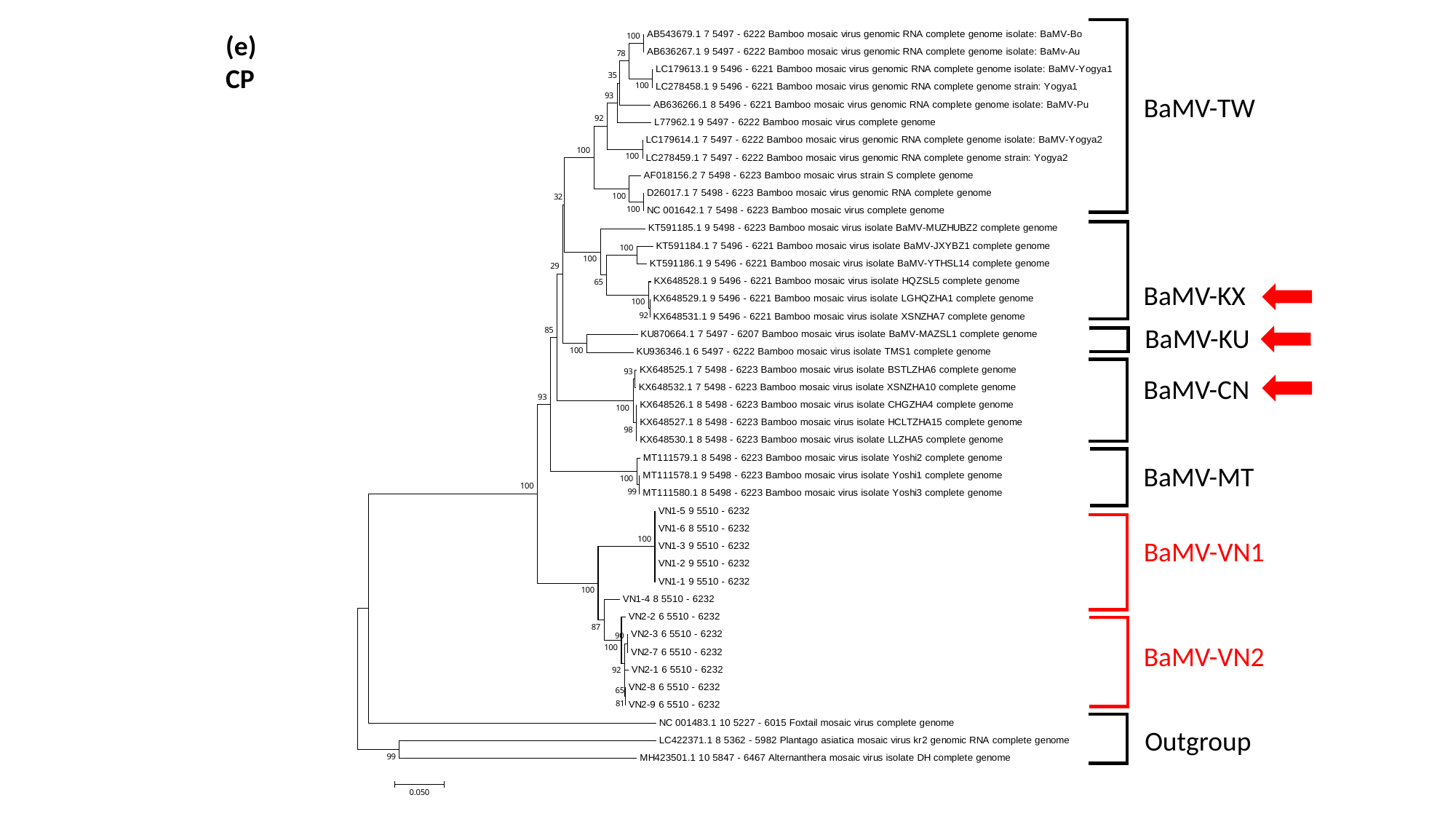

(e)
CP
BaMV-TW
BaMV-KX
BaMV-KU
BaMV-CN
BaMV-MT
BaMV-VN1
BaMV-VN2
Outgroup

## Slide 6
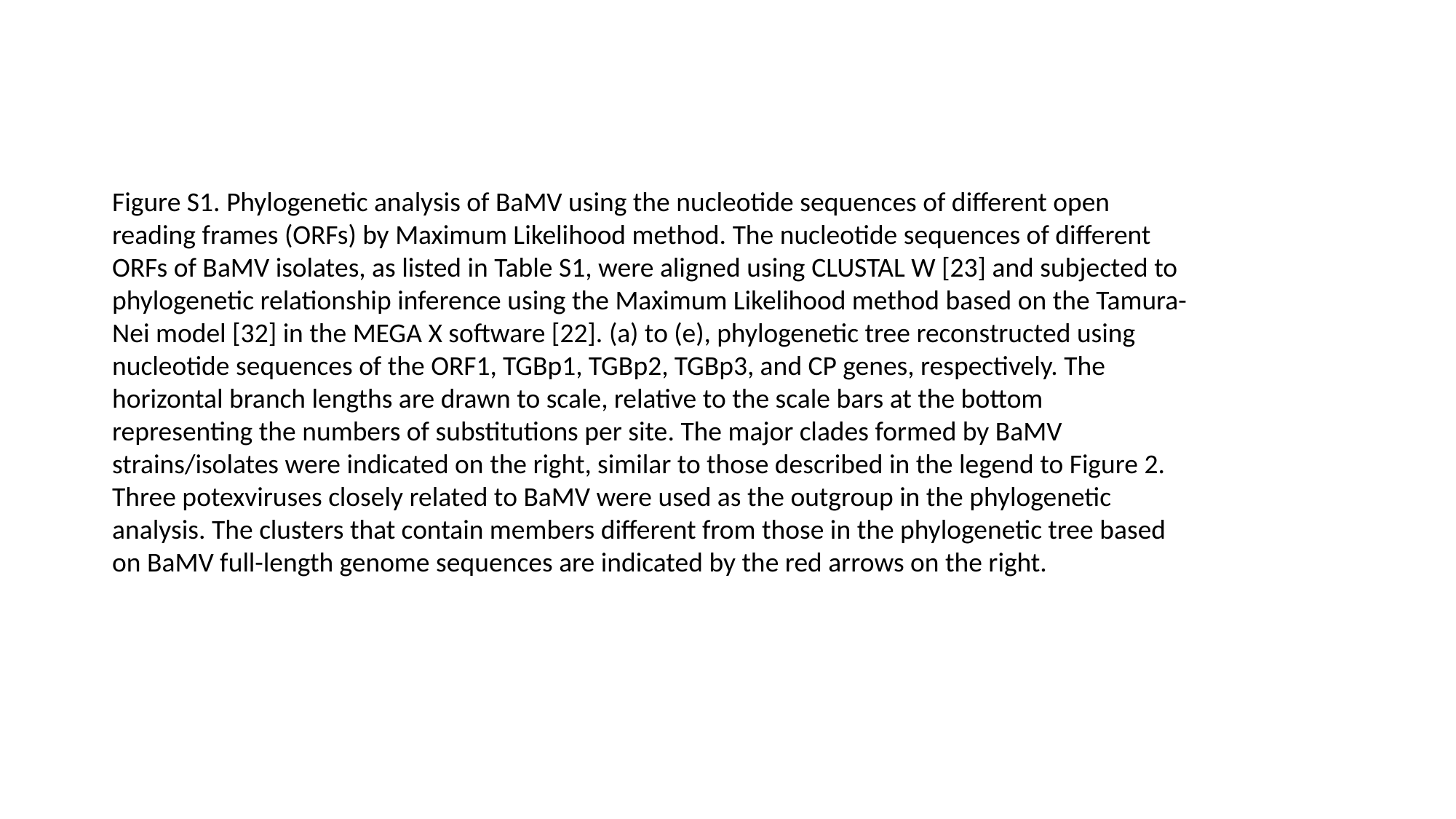

Figure S1. Phylogenetic analysis of BaMV using the nucleotide sequences of different open reading frames (ORFs) by Maximum Likelihood method. The nucleotide sequences of different ORFs of BaMV isolates, as listed in Table S1, were aligned using CLUSTAL W [23] and subjected to phylogenetic relationship inference using the Maximum Likelihood method based on the Tamura-Nei model [32] in the MEGA X software [22]. (a) to (e), phylogenetic tree reconstructed using nucleotide sequences of the ORF1, TGBp1, TGBp2, TGBp3, and CP genes, respectively. The horizontal branch lengths are drawn to scale, relative to the scale bars at the bottom representing the numbers of substitutions per site. The major clades formed by BaMV strains/isolates were indicated on the right, similar to those described in the legend to Figure 2. Three potexviruses closely related to BaMV were used as the outgroup in the phylogenetic analysis. The clusters that contain members different from those in the phylogenetic tree based on BaMV full-length genome sequences are indicated by the red arrows on the right.
