# Supplementary material for: First Report of Distinct Bamboo mosaic virus (BaMV) Isolates Infecting Bambusa funghomii in Vietnam and the Identification of a Highly Variable Region in the BaMV Genome
Source: Viruses. 2022 Mar 28;14(4):698. doi: 10.3390/v14040698 (PMC9032891; doi:10.3390/v14040698)
Supplement: Supplementary file 1 [file Table S1.pdf › Table S1.pdf]

Table S1. Percent nucleotide sequence identities between a selected *Bamboo mosaic virus* Vietnam isolate (BaMV-VN2-9) and other known BaMV strains in different regions.

Accession No.	Name	Origin	Full	5′UTR	ORF1	TGBp1	TGBp2	TGBp3	CP	3′UTR
**AB543679.1**	BaMV-Bo	Taiwan	77.3	84.9	76.4	77.3	78.8	69.9	80.2	94.4
**AB636266.1**	BaMV-Pu	Taiwan	76.6	84.9	75.7	76.7	78.1	70.7	78.5	93.8
**AB636267.1**	BaMV-Au	Taiwan	77.1	84.9	76.1	77.1	78.8	69.9	79.9	94.4
**AF018156.2**	BaMV-S	Taiwan	76.3	81.9	75.5	75.9	77.7	73.1	78	94.4
**D26017.1**	BaMV-O	Taiwan	76.5	83.3	75.7	76.3	76.9	73.8	78.2	94.4
**KT591184.1**	BaMV-JXYBZ1	Fujian	77.4	84	76.7	78.2	78.3	72.2	77	95.1
**KT591185.1**	BaMV-MUZHUBZ2	Fujian	77	85.1	76.2	77.1	78.4	73.2	77.8	96.5
**KT591186.1**	BaMV-YTHSL14	Fujian	77.9	85.1	77	78.3	79.4	74.1	78.5	95.1
**KU870664.1**	BaMV-MAZSL1	Fujian	78.1	84	77.8	77.5	78.8	70.6	78.9	94.4
**KU936346.1**	BaMV-TMS1	Fujian	77.2	85.1	76.4	77.4	79.1	69.6	79	93.8
**KX648525.1**	BaMV-BSTLZHA6	Fujian	77.4	84.4	76.8	77	77.3	70.9	79	93.1
**KX648526.1**	BaMV-CHGZHA4	Fujian	77.3	84.4	76.8	76.2	77.2	71.1	79.3	93.1
**KX648527.1**	BaMV-HCLTZHA15	Fujian	77.3	84.4	76.8	76.2	77.2	71.1	79.3	93.1
**KX648528.1**	BaMV-HQZSL5	Fujian	76.9	86.2	76.1	77	80	63.1	79.7	95.8
**KX648529.1**	BaMV-LGHQZHA1	Fujian	77	87.2	76.2	77.3	80	63.6	79.4	95.8
**KX648530.1**	BaMV-LLZHA5	Fujian	77.3	84.4	76.8	76.2	77.2	71.1	79.3	93.1
**KX648531.1**	BaMV-XSNZHA7	Fujian	76.9	86.2	76.1	77.1	80	63.6	79.4	95.8
**KX648532.1**	BaMV-XSNZHA10	Fujian	77.4	84.4	76.8	77	77.3	70.9	79.1	93.1
**L77962.1**	BaMV-V	Taiwan	77.2	83.9	76.5	75.3	79.9	75.6	79.2	95.1
**LC179613.1**	BaMV-Yogya1	Indonesia	76.3	84.9	75.4	76.7	78	70.1	77.9	95.8
**LC179614.1**	BaMV-Yogya2	Indonesia	76.9	84.9	75.6	77.6	79.9	73.3	80.2	93.8
**LC278458.1**	strain: Yogya1	Indonesia	76.3	84.9	75.4	76.7	78	70.1	77.9	95.8
**LC278459.1**	strain: Yogya2	Indonesia	76.9	84.9	75.5	77.6	79.9	73.3	80.2	93.8
**LC422371.1**	PlAMV	Korea	51.1	42	54.2	49.4	46.2	26.1	44.4	33.3
**MH423501.1**	AltMV	USA	49.9	43	51.7	53.3	45.8	38.1	41.1	39
**MT111578.1**	BaMV-Yoshi1	USA	76.7	84.7	75.1	78.8	79	78.5	78.6	97.2
**MT111579.1**	BaMV-Yoshi2	USA	77.1	84.7	75.5	78.8	81.3	79.1	78.6	97.2
**MT111580.1**	BaMV-Yoshi3	USA	76.9	84.7	75.4	78.6	78.7	78.5	78.6	97.2
**NC_001483.1**	FoMV	Canada	56.5	55.4	60.5	49.9	48.8	50.3	44.4	51.2
**NC_001642.1**	BaMV-O(Ref)	Taiwan	76.5	83.3	75.7	76.3	76.9	73.8	78.2	94.4
**VN1-1**	BaMV-VN1-1	Vietnam	95.9	98.9	95.6	95.8	96.6	94.9	96.8	100
**VN1-2**	BaMV-VN1-2	Vietnam	95.9	98.9	95.5	95.8	96.6	94.9	96.8	100
**VN1-3**	BaMV-VN1-3	Vietnam	95.9	98.9	95.6	95.8	96.6	94.9	96.8	100
**VN1-4**	BaMV-VN1-4	Vietnam	96	98.9	95.6	96.1	96.6	83.6	96.8	99.3
**VN1-5**	BaMV-VN1-5	Vietnam	95.8	98.9	95.5	95.8	96.4	92.9	96.8	100
**VN1-6**	BaMV-VN1-6	Vietnam	95.3	97.8	94.7	95.7	96.6	94.9	96.7	100
**VN2-1**	BaMV-VN2-1	Vietnam	99.9	100	100	100	99.7	100	99.6	99.3
**VN2-2**	BaMV-VN2-2	Vietnam	99.7	100	99.9	99.5	98.6	100	99.2	100
**VN2-3**	BaMV-VN2-3	Vietnam	100	100	100	100	100	100	99.6	99.3
**VN2-7**	BaMV-VN2-7	Vietnam	100	100	100	100	100	100	99.6	99.3
**VN2-8**	BaMV-VN2-8	Vietnam	100	100	100	100	100	100	100	99.3
**VN2-9**	BaMV-VN2-9	Vietnam	100	100	100	100	100	100	100	100
